# Investigating the relationship between hippocampus/dentate gyrus volume and hypothalamus metabolism in participants with major depressive disorder

**DOI:** 10.1038/s41598-024-61519-z

**Published:** 2024-05-09

**Authors:** Karen Lin, Daniel Sunko, Junying Wang, Jie Yang, Ramin V. Parsey, Christine DeLorenzo

**Affiliations:** 1 Cornell University, Ithaca, NY USA; 2 Stony Brook University, New York, NY USA; 3 Department of Applied Mathematics and Statistics, Stony Brook University, New York, NY USA; 4 Department of Family, Population & Preventive Medicine, Stony Brook University, New York, NY USA; 5 Department of Psychiatry and Behavioral Health, Stony Brook University, Stony Brook, NY USA; 6 Department of Biomedical Engineering, Stony Brook University, Stony Brook, NY USA

**Keywords:** Magnetic resonance imaging, Positron-emission tomography

## Abstract

Reduced hippocampal volume occurs in major depressive disorder (MDD), potentially due to elevated glucocorticoids from an overactivated hypothalamus–pituitary–adrenal (HPA) axis. To examine this in humans, hippocampal volume and hypothalamus (HPA axis) metabolism was quantified in participants with MDD before and after antidepressant treatment. 65 participants (n = 24 males, n = 41 females) with MDD were treated in a double-blind, randomized clinical trial of escitalopram. Participants received simultaneous positron emission tomography (PET)/magnetic resonance imaging (MRI) before and after treatment. Linear mixed models examined the relationship between hippocampus/dentate gyrus volume and hypothalamus metabolism. Chi-squared tests and multivariable logistic regression examined the association between hippocampus/dentate gyrus volume change direction and hypothalamus activity change direction with treatment. Multiple linear regression compared these changes between remitter and non-remitter groups. Covariates included age, sex, and treatment type. No significant linear association was found between hippocampus/dentate gyrus volume and hypothalamus metabolism. 62% (38 of 61) of participants experienced a decrease in hypothalamus metabolism, 43% (27 of 63) of participants demonstrated an increase in hippocampus size (51% [32 of 63] for the dentate gyrus) following treatment. No significant association was found between change in hypothalamus activity and change in hippocampus/dentate gyrus volume, and this association did not vary by sex, medication, or remission status. As this multimodal study, in a cohort of participants on standardized treatment, did not find an association between hypothalamus metabolism and hippocampal volume, it supports a more complex pathway between hippocampus neurogenesis and hypothalamus metabolism changes in response to treatment.

## Introduction

Major Depressive Disorder accounts for 10.3% of all disease burden worldwide^1^. MDD’s prevalence is partially driven by a lack of knowledge regarding its pathophysiology, hindering treatment development. As stressful life events often precede depression, examining the brain’s stress response, specifically the hypothalamus in the hypothalamic–pituitary–adrenal (HPA) axis, could shed light on the biology of MDD and antidepressant treatment response^2^.

Chronic stress results in an overactive HPA axis. Within minutes of a stressor, the HPA axis initiates a physiological response related to stress by stimulating parvocellular neurons in the paraventricular nucleus (PVN) of the hypothalamus. Corticotropin-releasing factor is released, which in turn induces the release of adrenocorticotropic hormone, responsible for glucocorticoid synthesis in the adrenal cortex^3^. Glucocorticoids, such as cortisol, range in function but play an important role in maintaining homeostasis after the stress stimulus. Specifically, to initiate the feedback loop that terminates the response, cortisol binds to glucocorticoid receptors (GR)^4,5^. The function of GRs, which provide negative feedback during stress^4^, can be disrupted by severe and prolonged stress. Prolonged exposure to cortisol also overworks immune responses and leads to the overproduction of proinflammatory cytokines. These proinflammatory cytokines influence GR function, typically by increasing expression of GRβ isoforms, which are inactive forms of GRs that compete with the active form GRα, causing glucocorticoid resistance^6^. The sensitivity of GRs is also regulated by the chaperone protein FKBP5. Transcription of FKBP5 increases with circulating corticosteroids through a hormone response element within a promoter of the gene^7,8^. FKBP5 binds to the GR complex, reducing its affinity for glucocorticoid binding, and decreasing the efficiency of GR translocation into the nucleus^9^. The overall effect of these events is to hamper negative feedback, resulting in an overactive HPA axis.

The hippocampus, within the limbic system, plays an important role in memory^10^ and decision making^11^. Reduced hippocampal volume is often associated with MDD^12^ and the glucocorticoid hypothesis suggests this may be due to a dysregulated HPA axis^13^. Extended exposure to glucocorticoids causes dendritic retraction in the hippocampus, a reversible form of volume loss that causes the hippocampus to be vulnerable to cell death^14^. Further, an animal model of an anxiety/depressive-like state suggests that cortisol hampers proliferation of progenitor cells in the hippocampus^15^. Partially supporting this glucocorticoid hypothesis, patients with depression report a higher rate of stressful life events^16^ and long-term stress is associated with volume loss in the hippocampus^17^. A model used to study the glucocorticoid hypothesis is Cushing’s syndrome, where glucocorticoids are commonly overproduced, which is often coupled with reduced hippocampal volume^18^. This may be a particularly relevant model because MDD accompanies Cushing’s syndrome in 51–81% of cases^19^.

One study showed that reducing cortisol levels in Cushing’s syndrome resulted in normalization of hippocampus volume, suggesting a reversible volume decrease^20^. Relatedly, a neurogenic hypothesis posits that neurogenesis is needed for recovery for MDD^21^. Correspondingly, selective serotonin reuptake inhibitors (SSRIs), the most commonly prescribed antidepressant therapy^22^, have been shown to normalize HPA activity, resulting in lower glucocorticoid levels in rats^23^ and increased posterior hippocampal volume in human participants^24^. Rodent models and retrospective analyses using post-mortem tissue suggest this neurogenesis occurs in the dentate gyrus of the hippocampus^25^, an area uniquely capable of adult neurogenesis that shows an increase in neural progenitor cells following antidepressant treatment^26^. Although separate studies have shown normalization of the HPA axis and an increase in hippocampus volume with antidepressant treatment, to our knowledge, no study has examined the relationship of HPA axis activity to hippocampal volume in humans.

As described above, PVN neurons within the hypothalamus are responsible for secretion of cortisol following stress and therefore the stress response is associated with increased activity of these neurons^27,28^. While the PVN neurons are a subcomponent of the whole hypothalamus, ex vivo cFos analysis has shown that the activation area of the hypothalamus increases with more aversive stressors^29,30^. As such, metabolism within the hypothalamus may serve as a proxy for HPA axis activity. This metabolism can be measured by 2-[^18^F]-fluorodeoxyglucose Positron Emission Tomography, FDG-PET. As validation of this measure in vivo, a positive correlation between hypothalamic blood flow (i.e. hypothalamic activity) and stress-induced cortisol level has been previously reported in humans^31^. In schizophrenia, treatment-induced changes in hypothalamic glucose metabolism measured by FDG-PET were significantly correlated with changes in plasma cortisol^32^. Similarly, in rats, an FDG microPET study found activation of the hypothalamus in response to acute stress, which terminated during recovery from the stressor^33^. Another FDG microPET study in rats showed that induced immobilization stress increased hypothalamus metabolism while coping methods both helped prevent the metabolism changes in the hypothalamus as well as the release of corticosteroids^34^. In this way, FDG-PET has been used to quantify the stress response of the HPA axis.

This study will compare hippocampal volume, including volume of the dentate gyrus specifically (as measured by MRI) to HPA axis metabolism (as measured by FDG-PET metabolic rate of glucose uptake in the hypothalamus) in participants with MDD, both pre- and post-treatment (placebo-controlled, randomized, double-blind treatment trial of escitalopram). It is hypothesized that there will be an inverse relationship between hypothalamus metabolism and hippocampal/dentate gyrus volume. The study will also examine the relationship between changes in hypothalamus activity and hippocampal size with treatment. It is hypothesized that a decrease in hypothalamus metabolism is required for hippocampal/dentate gyrus volume increase with treatment and is more likely to be observed in those who remit following treatment (regardless of treatment with escitalopram or placebo) than those who did not. It is a secondary analysis of a cohort with MDD designed to predict antidepressant treatment response from imaging^35^.

In addition, this study will examine whether these relationships differ between males and females. Differences in estrogen, progesterone, and testosterone levels can affect glucocorticoid feedback^36^. In female rats, a less robust negative feedback in the HPA axis and more dysregulation was associated with lower densities of mineralocorticoid receptors (MRs) and GRs and less glucocorticoid binding in the hypothalamus^37^. Compared to male mice, female mice also exhibited reduced innervation of the PVN^38^, and differing neuroplastic changes following stress including a reduction in synaptic input into stress-regulating regions not observed in male mice^39^. In a large sample of young adults, a more rapid ascent and decline of the HPA axis activity observed in males compared to females (all in the follicular phase to control for progesterone levels) indicated a healthier glucocorticoid-mediated negative feedback circuit^40^. In addition, PET studies showed that females have higher hypothalamus metabolism than men^41,42^. Thus, we hypothesized that hypothalamus metabolism will be higher in females and the relative volume of the hippocampus will also be smaller in females.

## Methods

This study, which was approved by the Institutional Review Board of Stony Brook University, included 85 outpatient participants with MDD who received pretreatment imaging (see CONSORT diagram in ref.^35^). All research protocols were performed in accordance with relevant regulations and guidelines. The study “Advancing Personalized Antidepressant Treatment Using PET/MRI” is registered on ClinicalTrials.gov (Registration Number: NCT02623205). The first registration was on 07/12/2015 and follow-up on the last participant was completed on 04/03/2020. Sample size was determined by power analysis to detect a true correlation in the primary outcome (see ref.^35^) as low as 0.39, with 80% power (two-tailed analysis, alpha = 0.05). Of the 85 participants, 65 participants (*n* = 24 males, *n* = 41 females, *n* = 31 in the escitalopram cohort, demographic and clinical information is provided in Table 1) were included in this study. Participants were not included if (*n* = 12) blood glucose measurements were not within 20% throughout the course of the PET imaging, (*n* = 7) they withdrew from the study or *n* = 1 had a diabetes diagnosis^35^. Of the 65 included participants, *n* = 1 pretreatment hippocampal/dentate gyrus volume was not used due to motion in the MRI, *n* = 1 pretreatment hypothalamus metabolism was not used due to motion in the PET imaging, and *n* = 2 received no or partial posttreatment imaging. All participants were at least 18 years old and were able to provide signed informed consent. All participants were verified by a trained rater as having been diagnosed with MDD, assessed via Structured Clinical Interview for DSM-IV (SCID-IV), and a score of 22 or higher on the Montgomery–Åsberg Depression Rating Scale^43^ (MADRS). The MADRS was used only for inclusion of participants and was not used as an outcome measurement in this study to prevent inflation of symptoms and artificial treatment response. Potential participants were excluded in the event that they had a significant active physical illness, were currently undergoing successful antidepressant treatment, had significant neurological deficits, had a high potential for excessive substance use during the study period, had electroconvulsive therapy within six months, had current psychosis, had a lifetime history of bipolar disorder, had medical contraindications to escitalopram (study drug), such as failed escitalopram therapy of appropriate dose and duration in the past, or had contraindications to MRI or PET imaging, such as pregnancy or breastfeeding. All participants were antidepressant medication-free for three weeks prior to the study, either having completed medication washout (for ineffective medication) or by enrolling as psychotropic medication free. PET/MRI data from this cohort has been published in previous studies^35,44–47^. None have examined the relationship between PET and MRI measures examined in this work.

**Table 1:** Descriptive table for each variable by medication type; Median + /– interquartile range are reported.

Variable	Total	Escitalopram	Placebo	*p*-value
Age (years) (n = 65)	23.3 ± 12.2	23.1 ± 27.3	23.5 ± 8.6	0.47
Sex: female (n = 41)	41 (63.1%)	19 (46.3%)	22 (53.7%)	0.78
Hippocampus volume pre (mm^3^) (n = 64)	8514.2 ± 1185.5	8414.1 ± 868.9	8753.5 ± 1314.8	0.25
Hippocampus volume post (mm^3^) (n = 64)	8461.5 ± 1098.0	8318.4 ± 1035.2	8554.1 ± 1489.9	0.35
Hippocampus volume change (%) (n = 63)	− 0.4 ± 3.6	− 0.4 ± 3.5	− 0.5 ± 3.4	0.86
Dentate Gyrus Volume Pre (mm^3^) (n = 64)	1098.5 ± 210.8	1091.9 ± 188.8	1118.4 ± 227.5	0.49
Dentate gyrus volume post (mm^3^) (n = 64)	1097.8 ± 192.3	1090.8 ± 170.3	1107.7 ± 234.3	0.33
Dentate gyrus volume change (%) (n = 63)	0.0 ± 7.5	− 0.1 ± 7.4	0.9 ± 8.5	0.93
Hypothalamus metabolism pre (mg/min*100 ml) (n = 64)	2.6 ± 0.4	2.7 ± 0.5	2.5 ± 0.4	0.18
Hypothalamus metabolism post (mg/min*100 ml) (n = 62)	2.5 ± 0.5	2.5 ± 0.5	2.6 ± 0.5	0.28
Hypothalamus metabolism change (%) (n = 61)	− 3.0 ± 24.0	− 6.1 ± 20.4	− 0.3 ± 22.4	0.10
Change in depression (%) (n = 65)	− 44.4 ± 44.3	− 44.4 ± 43.5	− 44.2 ± 42.9	0.59

Pre: before treatment; post: after treatment.

Prior to, and following approximately 8 weeks (60 ± 9 days) of treatment, simultaneous PET/MRI imaging was acquired on a 3 T Siemens Biograph mMR (Siemens, Erlangen, Germany) with a 12-channel head coil for 60 min. The average time of imaging was 11:53 AM ± 97.4 min. (FDG-PET measurements remain relatively consistent throughout the day while the person is awake^48,49^). Eight weeks of treatment was selected to ensure a sufficient duration had elapsed to allow participants to achieve remission while avoiding an extended period without medication in the placebo group. On average, it requires approximately six weeks of SSRI treatment to achieve remission^50,51^. A remission rate of 89.8% (response rate of 95.4%) after six weeks has been reported for escitalopram specifically^52^. Depression severity ratings occurred within six days of imaging except for two participants (8 and 9 days, average across all participants and pre- and post-treatment imaging: 0.6 ± 1.3 days). Treatment was initiated following imaging and ratings. Through a double-blind design, participants were randomized to treatment with either placebo or escitalopram. Escitalopram was chosen because of its high selectivity for the serotonin transporter compared with other FDA-approved SSRIs, wide usage, and efficacy in MDD^53,54^. It has been shown to be significantly more effective^55^ well-tolerated^56^ and have a faster onset of treatment effect than citalopram^57,58^. Group allocation for all participants was determined at study initiation by pseudo-random allocation scheme (1:1 ratio) generated by the pharmacist with the software Research Randomizer (http://www.randomizer.org/). All participants received the same number of pills (one pill per 10 mg of escitalopram in the escitalopram arm). Participants received 10 mg of escitalopram in week 1, 20 mg in weeks 2 and 3, and 30 mg in weeks 4–8, altered as needed for tolerance. All participants reached 30 mg (or 3 placebo pills) by week 8. Remission was defined a priori as post-treatment Hamilton Depression Rating Scale (HDRS-17) less than or equal to 7^35^.

### Magnetic resonance imaging (MRI)

A magnetization-prepared rapid gradient-echo (MP-RAGE) T1-weighted structural image was acquired, simultaneously with the PET imaging, with the following parameters: TR = 2300 ms, TE = 3.24 ms, flip angle = 9 degrees, IPAT GRAPPA factor 2, FOV = 223 × 210x195mm, bandwidth = 220 Hz/Px, echo spacing = 7.8 ms, voxel size = 0.87 × 0.87 × 0.87 mm. T1 structural images were processed through the automated hippocampal subfield segmentation pipeline of Freesurfer 5.3.0 (http://surfer.nmr.mgh.harvard.edu) to automatically extract the dentate gyrus as well as the whole hippocampus from the Desikan-Killiany atlas^59,60^. The hypothalamus was delineated via nonlinear registration of the participant’s T1 image to the MNI template for the use of the CTI168 high-resolution subcortical brain nucleus atlas^61,62^. Nonlinear warp parameters were generated using Advanced Normalization Tools (ANTs)^61,62^. For quality control, an overlay of the warped region on each participant’s MRI was visually inspected.

### Positron emission tomography (PET)

PET images were collected for 60 min. Raw listmode PET data were reconstructed offline using Siemens’ e7 Tools software and a CT-like Boson MR-based attenuation map^63,64^. Sinogram files were generated using the following frame definitions: 8 × 15 s, 6 × 30 s, 5 × 60 s, 4 × 300 s, and 3 × 600 s. Frames were corrected for motion^65^ and co-registered to the MRI. The hypothalamus regional delineation was transferred to the PET images through the co-registration. The Patlak graphical approach was used to estimate metabolic rate of glucose uptake (MRGlu) from the time activity curve while correcting for blood glucose and the lumped constant, using Simultaneous Estimation and a single venous sample, as previously described^35^.

### Statistical analysis

Escitalopram and placebo subgroups were examined together, as multiple studies have reported similar treatment responses^66–70^. And, neurobiological changes quantified by FDG-PET in were not different across patients who achieved depression remission using either placebo or SSRI^71^. However, treatment status was used as a covariate in all analyses.

Linear mixed models were utilized to examine the relationships between hippocampus/dentate gyrus volume and hypothalamus metabolism with age, sex, and pre/post-treatment timepoint as covariates. To account for differences in intracranial volume (ICV), normalized hippocampus/dentate gyrus volumes (volume*100/ICV) were also analyzed in the model^72^. Two-way interactions between sex and hypothalamus metabolism as well as between pre- and post-treatment timepoint and hypothalamus metabolism were further examined to model the sex-specific relationships and pre- and post-specific relationships in separate models. For the hippocampus, Unstructured variance–covariance structures were selected with a smaller Akaike Information Criteria (AIC) than Compound Symmetric. For the dentate gyrus, Compound Symmetric variance–covariance structures were selected with a smaller AIC than Unstructured.

A chi-squared test with exact *p*-values based on Monte Carlo simulation was used to examine the marginal association between the categorical variable (sex) and treatment cohorts (medication type: escitalopram, placebo) as well as between hippocampal/dentate gyrus volume change direction (increase or decrease) and hypothalamus metabolism change direction (increase or decrease). (If HPA Axis does regulate hippocampal volume, hypothalamus activity decreases should be associated with hippocampal volume increases and vice versa.) Wilcoxon rank sum tests were used to compare unadjusted marginal differences for any continuous covariates (age, hypothalamus metabolism before and after treatment, percent change in hypothalamus metabolism, and percent change in depression) as well as continuous outcome variables (hippocampus/dentate gyrus volume before and after treatment and percent change in hippocampus/dentate gyrus volume) among the two groups (escitalopram, placebo). Spearman rank correlation coefficient was used to measure the linear relationships between the percent change in hippocampus/dentate gyrus volume and percent change in hypothalamus metabolism/percent change in depression by medication type. A multivariable logistic regression model was performed to model the association between hypothalamus metabolism change direction and hippocampus/dentate gyrus volume change direction, adjusting for age, sex, and medication type. (This association was examined as another way of validating the hypothesis that the change hypothalamus activity should be inversely correlated to change in hippocampus volume with treatment.) A two-way interaction between sex and hypothalamus metabolism change was further examined to model the sex-specific relationship.

Multiple linear regression models with percent change in hypothalamus activity interacted with remission status were implemented to compare the differences in the relationships between percent change in hypothalamus activity and hippocampus/dentate volume between remitter and non-remitter groups; as well as with percent change in depression interacted with medication type to evaluate the medication type-specific relationships to the percent change in hypothalamus activity/ hippocampus volume/dentate gyrus volume, adjusting for age and sex.

Statistical analysis was performed using SAS 9.4 and significance level was set at 0.05 (SAS Institute Inc., Cary, NC).

## Results

As displayed in Table 1, none of the examined outcome measures (hippocampus volume, hypothalamus metabolism) or covariates (age, sex) were statistically significantly different between the active medication and placebo groups. For this reason, they are combined in subsequent analyses (though treatment type remains as a covariate).

### Relationship between hypothalamus metabolism and hippocampal volume

There was no strong evidence for a significant linear relationship between hippocampus volume and hypothalamus metabolism (estimated coefficient = − 56.5, 95% CI [− 231.0, 118.0], *p*-value = 0.52), adjusting for age, sex, and pre- and post-treatment timepoint. Female participants had significantly lower hippocampus volume than male participants (*p-*value < 0.01), adjusting for age, hypothalamus metabolism and pre- and post-treatment timepoint. Hippocampus volume had a significantly negative relationship with age (*p*-value < 0.01), adjusting for sex, hypothalamus metabolism and pre- and post-treatment timepoint. There was no significant difference between female and male hypothalamus metabolism (*p-*value = 0.11), while a significantly negative relationship with age was also found (*p*-value = 0.01). Results were similar when normalizing by intracranial volume (non-significant linear relationship between normalized hippocampus volume and hypothalamus metabolism [*p*-value = 0.24] and female participants had reduced normalized hippocampal volume [*p*-value = 0.001], although a non-significant relationship between normalized hippocampal volume and age was found [*p*-value = 0.28]).

Similarly, no strong evidence existed to show a significant linear relationship between dentate gyrus volume and hypothalamus metabolism (estimated coefficient = − 8.7, 95% CI [− 40.9, 23.6], p-value = 0.59), adjusting for age, sex and pre- and post-treatment timepoint. Female participants had significantly lower dentate gyrus volume than male patients (*p*-value < 0.01), adjusting for age, hypothalamus metabolism and pre- and post-treatment timepoint. However, dentate gyrus volume was not significantly associated with age (*p*-value = 0.14), adjusting for sex, hypothalamus metabolism and pre- and post-treatment timepoint. Results were similar when normalizing by intracranial volume (non-significant linear relationship between normalized dentate gyrus volume and hypothalamus metabolism [*p*-value = 0.50], although non-significant relationships between normalized dentate gyrus volume and both sex [*p*-value = 0.06] and age were found [*p*-value = 0.28]).

Neither of the two interaction terms, examined in separate linear mixed models, were significant, which indicated the linear relationship between hippocampus volume (or dentate gyrus volume) and hypothalamus metabolism was not significantly different by sex or time point (pre-treatment or post-treatment).

### Relationship between change in hypothalamus activity and change in hippocampal volume with treatment

Table 2 shows no strong evidence for a relationship between a change in hypothalamus metabolism and a change in hippocampal (or dentate gyrus) volume based on the Chi-squared test, meaning a decrease in hypothalamus metabolism was not associated with an increase in hippocampal (or dentate gyrus) volume. The multiple linear regression model also showed no significant linear relationship between percent change in in hypothalamus metabolism and percent change in hippocampus/dentate gyrus volume (hippocampus: estimated coefficient =  − 0.02, 95% CI [− 0.08, 0.04], *p*-value = 0.51; dentate gyrus: estimated coefficient = − 0.03, 95% CI [− 0.11, 0.06], *p*-value = 0.50) adjusting for age, sex and medication type. The results were unchanged in a subgroup analysis involving only those randomized to escitalopram (hippocampus: estimated coefficient = − 0.01, 95% CI [− 0.08, 0.06], *p*-value = 0.72; dentate gyrus: estimated coefficient = − 0.07, 95% CI [− 0.19, 0.05], *p*-value = 0.22) adjusting for age and sex).

**Table 2:** Univariate analysis between hippocampus (hip)/dentate gyrus (DG) volume change and hypothalamus metabolism change.

Variable	Change	Total	Hip volume change		*p*-value
			Decrease	Increase	
Hypothalamus metabolism change	Decrease	38 (62.3%)	21 (58.3%)	17 (68.0%)	0.59
	Increase	23 (37.7%)	15 (41.7%)	8 (32.0%)	

Variable	Change	Total	DG volume change		*p*-value
			Decrease	Increase	
Hypothalamus metabolism change	Decrease	38 (62.3%)	18 (60.0%)	19 (63.3%)	1.00
	Increase	23 (37.7%)	12 (40.0%)	11 (36.7%)	

However, as seen in Table 2, a slightly higher percentage of those who experienced a decrease in hypothalamus metabolism had an increase in hippocampal/dentate gyrus volume following treatment and a slightly higher percentage of those who experienced an increase in hypothalamus metabolism had a decrease in hippocampal volume. Based on this, an odds ratio (OR) was calculated based on different multivariable logistic regression models and it was found that participants with decreased hypothalamus metabolism were estimated to have a larger, but not statistically significant, chance of having an increase in hippocampus/dentate gyrus volume than participants with increased hypothalamus metabolism after adjusting for age, sex and medication type (hippocampus: OR = 1.87 > 1, 95% CI [0.6, 5.9], *p*-value = 0.28; dentate gyrus: OR = 1.12 > 1, 95% CI [0.4, 3.6], *p*-value = 0.85). The results were unchanged in a subgroup analysis involving only those randomized to escitalopram (hippocampus: OR = 1.52, 95% CI [0.29–7.98], *p*-value = 0.62; dentate gyrus: OR = 2.02, 95% CI [0.36–11.24], *p*-value = 0.42).

The sex interaction term was further examined using a multivariable logistic regression model and was not significant, indicating that the association between hippocampus/dentate gyrus volume change and hypothalamus metabolism change was not significantly different across sexes, adjusting for age and medication type as covariates. Similarly, in the multiple linear regression model, the linear relationship between percent change in hypothalamus metabolism and percent change in hippocampus/dentate gyrus volume was not significantly different by sex, adjusting for age and medication type as covariates.

### Relationship between hippocampus volume and hypothalamus activity in remitters versus non-remitters

The relationship between percent change in hypothalamus metabolism and percent change in hippocampus/dentate gyrus volume did not differ significantly by remission status (hippocampus: *p*-value = 0.87, dentate gyrus: *p*-value = 0.42, Fig. 2).

For completeness, the relationship between hypothalamus metabolism and hippocampal/dentate gyrus volume was examined separately pre- and post-treatment. No significant linear relationship was found in remitters or non-remitters (and there were no significant differences in this relationship between the two groups) with or without normalizing by intracranial volume.

## Discussion

Chronic stress is associated with both increased HPA activity and reduced hippocampal volume, though these have not been quantified within the same individual. Our study sought to pinpoint the relationship between HPA activity (as assessed by hypothalamus metabolism) and hippocampal volume by examining several measures: the correlation between hypothalamus metabolism and hippocampal volume within an individual, the relationship between change in hypothalamus activity and change in hippocampal volume (Chi squared and multiple linear regression) with treatment, the odds ratio of direction of change of hippocampal volume given the direction of change of hypothalamus activity with treatment, and percent change of hypothalamus activity / hippocampal volume in depression remitters versus non-remitters.

The dentate gyrus within the hippocampus was examined because this is the site of hippocampal neurogenesis^24^. Examining dentate gyrus volume may therefore provide higher resolution than examining hippocampus volume as a whole. However, as can be seen in Fig. 2, the range of percent differences with treatment are similar. Relatedly, the volume of the dentate gyrus is significantly correlated with total hippocampal volume both before (*r* = 0.91, *p*-value < 0.0001) and after treatment (*r* = 0.92, *p*-value < 0.0001).

**Figure 1:**
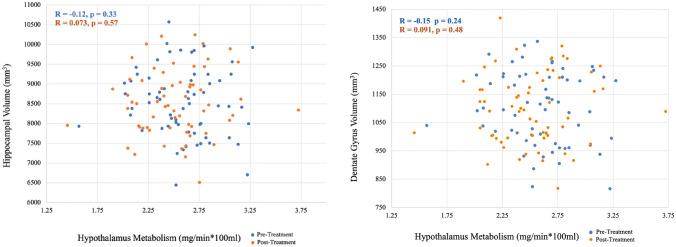
Hippocampus (Left) or dentate gyrus (Right) volume versus hypothalamus metabolism including pre-treatment (blue) and post-treatment (orange).

**Figure 2:**
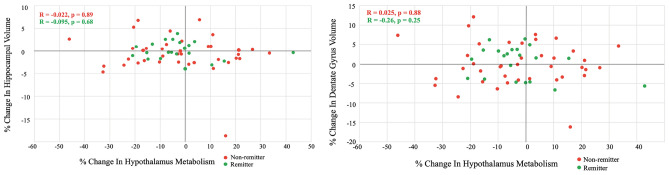
Percent change in hippocampus (Left) or dentate gyrus (Right) volume versus percent change in hypothalamus metabolism including non-remitters (red) and remitters (green).

As the scatterplots in Fig. 1 reveal, no association between hippocampus/dentate gyrus volume and hypothalamus metabolism was observed, before or after treatment or in the combined cohort. In Table 2, it was shown that, following treatment, although participants who experienced a decrease in hypothalamus metabolism had a higher chance of hippocampus/dentate gyrus volume increase than participants with an increase in hypothalamus metabolism, the result was not statistically significant. The cohort was then divided into those who remitted and those who did not (Fig. 2) in case the relationship was only apparent in those who recovered from the treatment. However, when separating remitters and non-remitters, still no significant relationship was found. In a separate analysis (data not shown), there was also no strong evidence of a linear relationship between percent change in hypothalamus metabolism or hippocampal/dentate gyrus volume and percent change in depression, adjusting for age, sex, and medication type.

Although not evident in this study, it is possible that the relationship between hypothalamus activity and hippocampal volume exists as hypothesized, but a larger sample size is required to detect it. Given that the current sample contains > 60 participants, this suggests the effect size may be too weak to be clinically relevant. Rather, it is more likely that the consistent evidence above, examined in multiple ways using multiple modalities, suggests that the relationship between hypothalamus activity and hippocampus volume is complex and/or may not be linear. For example, it is possible that the hippocampal size is related to factors other than, or, in addition to HPA axis activity (see “Limitations”). These factors could include inflammation or even serotonin-mediated pathways independent of hypothalamus activation. Regarding the former, stress induced mice showed neuroinflammation from microglial cell releasing a surge of proinflammatory cytokines^73^. Microglial cells secrete a variety of harmful non-discriminative factors resulting in reduction in neurogenesis and neuronal damage^74^. Related to the latter, patients with MDD may show reduced brain serotonin mediated neurogenesis. SSRI treatment may affect this serotonin-mediated pathway independent of hypothalamus activation. As a significant increase in hippocampal/dentate gyrus size was not observed in this study, it is possible that these changes are influenced by all of these mechanisms or vary greatly between individuals.

Interestingly, on average, hypothalamus metabolism changes with treatment were higher than hippocampal volume changes. The ranges are: hippocampus (− 18.7 to 9.8%), dentate gyrus (− 16.2% to 12.1%), and hypothalamus metabolism (− 46.1 to 42.9%). Unexpectedly, hippocampus volume decreased, on average, with treatment in both active treatment and placebo groups. However, the average change was < 1% with a standard deviation of ~ 4%. Additionally, these differences were not significant. Although previous studies have reported volume increases over the course of treatment, it may be that metabolism changes occur more quickly and precede volume increases in this cohort, and that volume changes would occur over a longer period. This is consistent with previous analysis showing that most metabolic changes occur prior to those of structural^75^. However, considering that the treatment duration used may have been insufficient to detect significant differences in biological response, these findings focus on short-term correlations between hypothalamus and hippocampus changes and treatment response (Supplementary Information).

### Limitations

There are several limitations to this study: As the average age of our sample is 29.8 years (range: 18.2–64.5, median is 23.3 and IQR is 12.2), it is not a representative sample of the population with MDD. However, the age range is reflective of an important target population of this study since individuals 18–25 are reported to have the highest prevalence of MDD^55^. Though important covariates were included, depression is a heterogenrous illness and, due to power considerations, not all clinical variables could be included in the analyses. However, we have previously reported no significant differences in age of onset between remitters and non-remitters^35^ as well as age of onset and medication status between treatment and placebo cohorts^45^. Due to the tight age range of this cohort, age of onset is likely correlated with duration of illness and the number of depressive episodes. We further evaluated history of antidepressant use, number of antidepressant treatment trials, and MDD subtype and found no differences between between remitters and non-remitters or treatment and placebo cohorts (data not shown). Another limitation involves the use of hypothalamus metabolic rate of glucose uptake as a proxy for HPA axis activity. The most used method of HPA axis sampling is measuring cortisol in blood^27^. However, as mentioned above, hypothalamic activity and changes in this activity have been associated with cortisol level and changes in cortisol level in rodents and humans. Additionally, the HPA axis is directly and indirectly controlled by multiple brain regions. The dorsomedial and ventromedial hypothalamus are direct controls, but so are the ventrolateral medulla and the nucleus tractus solitarius. Indirect controls integrating their signals to activate the HPA axis include the medial prefrontal cortex, hippocampus, amygdala, and septum^27^. Therefore, examining the hypothalamus in isolation may not provide enough information for assessing HPA activity.

Despite the limitations, this study also had several strengths. A major strength of this study includes a rigorous statistical analysis, with multiple measures, of a large sample size of patients with MDD using PET and MRI. To our knowledge, no study has investigated the relationship between the hypothalamus activity and hippocampus volume utilizing the multimodal brain imaging techniques PET and MRI, allowing simultaneous assessment of both metabolism and structure, respectively. Additionally, use of hypothalamus activity prevents the need for self-report of stressful life events which may be subject to bias^76,77^. Our investigation also includes inclusion of intracranial volumes as well as covariates such as age and sex, with known effects on metabolism and volume, although we present no evidence to show that sex affects the relationship between hypothalamus metabolism and hippocampal volume. This is especially interesting given the known sex differences in these systems. Further, we provide insight into both hypothalamus metabolic and hippocampal volume changes with an active treatment (escitalopram) versus placebo. We observe no differences in neurobiological effects of these two treatments, even when accounting for remission status. This is consistent with our previous work showing changes in glucose metabolism across the brain were not significantly different between placebo and SSRI treatment^71^, as well as the results of other studies cited above. However, this is the first study, to our knowledge, to show a non-significant difference in the relationship between hypothalamus activity and hippocampal volume with treatment type, a significant finding given the purported relationship between HPA axis activity and hippocampal neurogenesis, and the importance of this circuitry in MDD and in response to treatment. Finally, results of our study provide a pathway for future research. Given the difficulty of replicating significant findings in biomedical research, the publication of negative results from well-powered studies is imperative for prevent overuse of resources in similar analyses^78^. Specifically, as this thorough, well-powered investigation failed to find a relationship between hypothalamus metabolism and hippocampal volume, future work should instead include a more comprehensive approach (e.g. regions in addition to the hypothalamus).

### Supplementary Information

Supplementary Information.

## Data Availability

The datasets used during this study is available from the corresponding author upon reasonable request.
